# A call for research and policy into building multi-layered social resilience toward a sustainable agricultural workforce in Switzerland

**DOI:** 10.3389/fpubh.2025.1617575

**Published:** 2025-08-14

**Authors:** Julia Doetzer, Andrea Kaiser-Grolimund, Priska Ammann, Medea Imboden, Ayoung Jeong, Aline Veillat, Karin Ingold, Mirko S. Winkler, Samuel Fuhrimann, Nicole Probst-Hensch

**Affiliations:** ^1^Department of Epidemiology and Public Health, Swiss TPH, Allschwil, Switzerland; ^2^Department of Public Health, University of Basel, Basel, Switzerland; ^3^Department of Anthropology, Durham University, Durham, United Kingdom; ^4^Institute of Political Science, University of Bern, Bern, Switzerland; ^5^Department Environmental Social Sciences, EAWAG, Dübendorf, Switzerland; ^6^Oeschger Center for Climate Change Research, University of Bern, Bern, Switzerland

**Keywords:** agriculture, social resilience, mental health, occupational health, Switzerland, multi-layered

## Abstract

The agricultural workforce is exposed to rapidly changing working conditions due to societal, economic, political, and ecological challenges. In the Swiss farming community, poor mental wellbeing is a growing concern and research focuses on the distribution and hazards of psychological distress in farmers and their social network. This perspective benefits from insights of the first agricultural cohort in Switzerland, illustrating the complex field that farmers operate in. Consequently, we call for a paradigm shift in research and policy from individual vulnerability to multi-layered social resilience toward building an agricultural workforce with the capacity to create pathways for a sustainable agriculture.

## Introduction

Swiss farmers operate in a field of competing views and interests. For example, the climate crisis increasingly threatens sustainable food production, while political and societal pressure grows to reduce farming’s environmental impact (1). Furthermore, sociopolitical goals in Switzerland are directly linked to requirements for farmers to obtain federal subsidies (2). Nevertheless, agricultural training in Switzerland continues to be popular (3). Yet, it is well known that stressful work conditions or poor societal recognition can adversely impact on workers’ vulnerability to poor mental health (4, 5). The federal competence center for agricultural research, *Agroscope*, provides evidence indicating higher rates of burnout among farmers compared to the general population (6). Moreover, male farmers in Switzerland show an elevated suicide rate compared to other men, with the gap widening (7). Reinforcing the evidence on Swiss farmer’s mental pressures, a descriptive overview of the FarmCoSwiss cohort, a quantitative health study among Swiss farmers from the three main language regions, revealed lower mental than physical health scores derived from the SF-12 v1, especially in younger age groups and women (8, 9). The assessment of flourishing in the context of farm characteristics and occupational hazards, revealed that farmers with the smallest farms (less than 5 hectares) scored lower in the financial and material stability domain than farmers with the largest farms (more than 50 hectares) (10, 11). A detailed description of the FarmCoSwiss methodology and descriptive baseline findings are available elsewhere (8, 11).

This perspective benefits from additional insights into farmer’s own views obtained in the FarmCoSwiss cohort of adult farmers and their partners working on the farm. It is mainly drawing from a free comment field in the baseline questionnaire (“If you would like to tell us anything else, you will find space here for suggestions, requests, comments or criticism.”). About 18% of all participants (*N*_Total_ = 872) shared their thoughts. Additionally, participants were asked at baseline for hypothetical reasons to choose or give up the job, respectively.

Based on the concerns farmers brought to the FarmCoSwiss study, we argue for a new research and policy focus on *multi-layered* social resilience in agricultural health for sustainable farming futures. Our call is aligned with early demands for more evidence on the resilience of farmers made by Fraser et al. in 2005 (12) but extends them from a focus on individual to multi-layered social resilience (5). Social resilience has been defined as “the capacity of actors to access capitals in order to – not only cope with and adjust to adverse conditions (that is, reactive capacity) – but also search for and create options (that is, proactive capacity), and thus develop increased competence (that is, positive outcomes) in dealing with a threat” (5), p. 289. Obrist et al. further acknowledge the multi-layered aspect, stating that “on each layer, but also across layers, actors are part of a social field that is defined with reference to the identified threat” (p. 290) (5, 13). Consequently, the concept of multi-layered social resilience extends beyond the immediate social circle of the individual farmer, encompassing various layers of society and the environment, including governmental regulations and laws.

## “More and more is demanded…”

**Figure 1:**
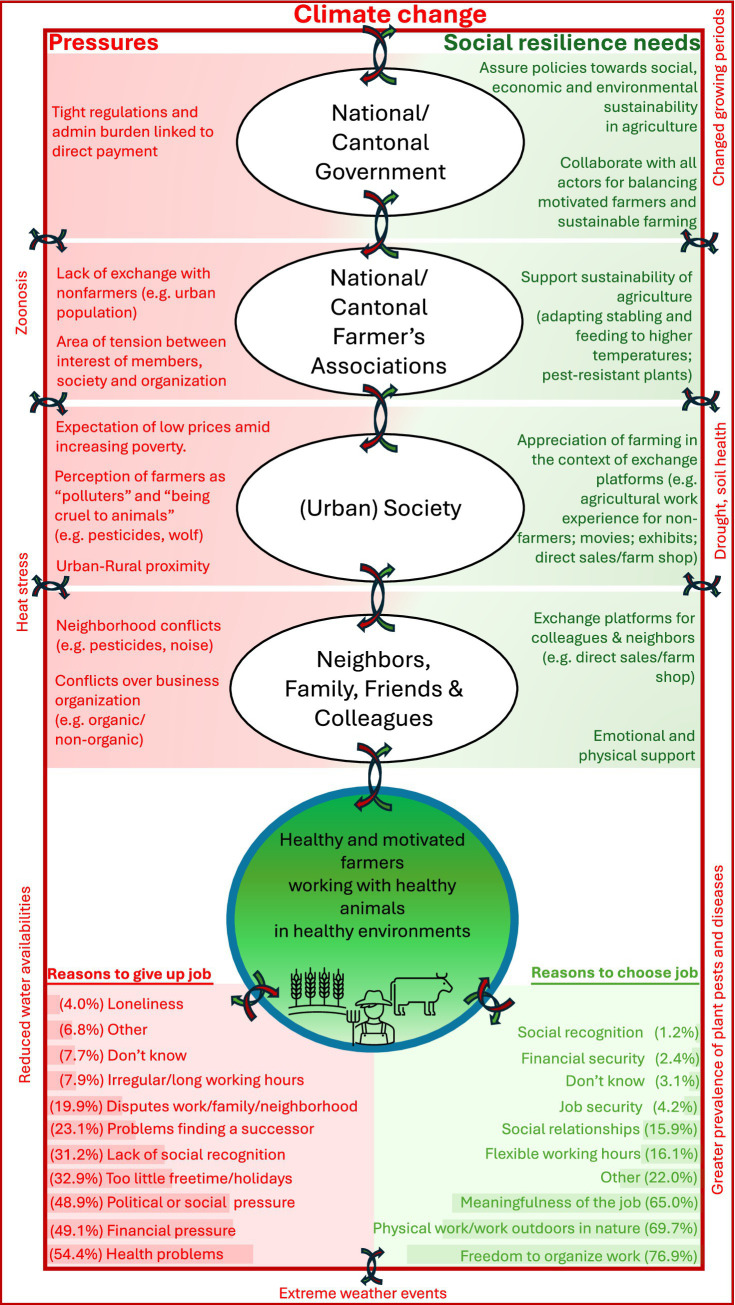
shows the conceptual framework (developed by authors, informed by Obrist et al. (5)) of multi-layered social resilience in the context of climate change. Red font and arrows illustrate potential ‘pressures’ or ‘risk factors’, while green font and arrows represent potential ‘social resilience needs’. The different layers are illustrated by ovals. Arrows between the layers represent interconnectedness. This illustration is not exhaustive and is intended to show what the different layers and factors within the Swiss farming context could look like, as exemplified by the issue of climate change. Reasons to give up job & reasons to choose job are retrieved from the FarmCoSwiss baseline survey.

When asked which three main reasons could hypothetically make farmers quit their job, besides health problems the most commonly selected reasons were financial, political, and societal pressures (Figure 1). Additionally, in the free comment field many participants commented on heavy workload and administrative burden. For example, Jennifer^1^ (69, organic^2^ farm), wrote: “[…] At the end of life, the realization comes that I’ve worked too much… Tragic - my pension goes toward the rent. I’ve worked so much and finally have to go to the social welfare office. That depresses me. […].” Non-organic^3^ farmer Martin (32) adds feelings of pressure by authorities: “In addition to the heavy workload from the hours worked and the great responsibility, this is becoming ever greater due to the authorities. More and more is demanded in the direct payment ordinance^4^ and the remuneration for this is becoming less and less. The authorities are also making it increasingly difficult to move the farm forward (building projects, reorganization, etc.) […].” Many participants also commented on the lack of recognition by society. Non-organic farmer James (49) wrote: “A major issue that concerns farmers is the recognition or non-recognition of their work within society and by consumers. You often have the feeling that you have to justify yourself for various work steps and activities. Farmers often have the feeling of being the boo man. […].” Moreover, organic farmer Jeremy (61) points out how positive job aspects can vanish due to societal changes: “The practice of therapeutic work with animals and in outdoor settings is being significantly undermined by the prevailing trends of modernity.”

## “Farming is not a profession, it is pure passion”

When asked which three main reasons would hypothetically make farmers choose their job again, they selected most often freedom to organize work, physical labor and meaningfulness of the job (Figure 1). The latter is reflected in many comments, including that of James (39, non-organic farming) cited in the title of this sub-section. Susan (47), organic farming, saw positive aspects besides physical constraints: “You learn to do your daily work despite physical limitations if you are satisfied, happy and in love with your job.” Similarly, Paul (61, organic farming) wrote: “I am convinced that the production of sustainable food is one of the most valuable jobs, and I am also prepared to work physically.” Other participants identified specific occupational benefits, including working with animals, colleagues, or customers. Thus, organic farmer Kristin (58) does not seem to feel a lack of recognition from customers: “Nevertheless, I am satisfied because I am doing something important and receive appreciation from my customers.” Moreover, Claudia (44, non-organic farming) says: “[…] But working with animals and nature gives you so much back and is still the most beautiful profession.” Echoing many positive aspects, Alexander (67) will continue to engage on a non-organic farm: “I’m still working past retirement age because I enjoy the people I work with and the work itself.”

## Toward multi-layered social resilience and sustainable farming

The concerns expressed by participants, such as high workload and financial pressure in the presence of effort-reward-imbalance, resonate contemporary evidence on job-specific mental wellbeing stressors (14). While it is important to understand causes and vulnerability factors of farmers’ stress, a focus shift toward social resilience would allow to identify enabling factors and capacities at multiple levels related to the agricultural sector. Individual resilience modifies the subjective experience of stress. Yet, coping mechanisms at the individual level may overwhelm individual resilience in the light of repeated and fast-changing challenges and limited social resilience.

An essential first step in researching multi-layered social resilience is clearly defining the threats according to Obrist et al. (5). Some of the threats are reflected in the farmer’s comments, but additional aspects and priorities need to be further explored. As a second step, the outcome of resilience building must be defined. From a farmer’s perspective, the goal may be the promotion of a workforce that is satisfied by its occupation and recognized for its work by society. From a broader agricultural and societal perspective, this workforce may be expected to contribute to sustainable food production, promote a healthy environment, and ensure responsible animal welfare. As a third step, it is essential to determine whether the objective is to build resilience “(pre-impact)” or manifest it “(post-impact),” as these are two different but equally important processes (5), p. 290.

To promote multi-layered social resilience and sustainable farming, we need to better understand the multiple constraints and enabling factors of not just the farmers themselves, but of the various actors involved. Sustainable farming thereby involves not only human actors but happens in a context that is entangled with animals and the environment. A holistic One Health approach (15) that takes these interconnections into account, may therefore benefit research on social resilience. The participants’ comments point to rewarding aspects at the individual level, such as caring for animals, nature, and customers.

At the level of farmer’s social network, research by the European Foundation for Improvement of Living and Working Conditions (Eurofound), discovered that social support among farmers is higher than in other occupations (16). Similarly, other studies suggest that family can be an important source of social support for farmers in times of crisis (17). With 79.3% the proportion of married FarmCoSwiss participants^5^ is particularly high, given that about 49% of people over 18 are married in the general population in Switzerland (18).

At the level of society and politics, members of the Swiss parliament with a link to farming account for roughly 16% of all seats (compared to 1.8% of the population that are farmers in Switzerland) (19). When FarmCoSwiss participants were asked at the first follow-up survey, answered by *N* = 600 participants on average 347 days after the baseline survey, how supported they felt on average by their cantonal and national farmers associations in regards to health, nearly 75% answered they felt moderately to fully supported^5^. However, whether and how this strong political representation benefits farmer’s mental health, wellbeing and resilience is yet to be explored.

Figure 1 illustrates a conceptual framework for multi-layered social resilience in the Swiss agricultural context, highlighting selected specific stressors and possible pathways toward a resilient agricultural workforce. This is further exemplified by a case vignette in Box 1. So far, much of the mental health research of farmers has focused on risk rather than enabling factors. In a review from 2019 about research trends in farmers mental health, Hagen et al. highlighted that only 5.9% of the identified studies (*N* = 341) focused on resilience (20). To our knowledge, there is no study applying a holistic multi-layered social resilience concept, as defined by Obrist et al. However, given the interconnectedness of the different layers, we argue that the lens of a holistic social resilience approach could benefit research on farmers’ mental health. Addressing this in a comprehensive inter- and transdisciplinary science-to-policy project would further enhance this approach. Concrete science and policy recommendations are summarized in Table 1.

**BOX 1 Case Vignette of farmer Max, illustrating different enabling factors and pressures.**

*The case of the 35-year-old farmer Max*

Max grew up on his parents’ farm. Two years ago, he and his wife and two children took over the farm, which had always been run non-organically. Believing that organic farming would better prepare him for the challenges of climate change, Max started to implement organic practices 2 years ago. This is a source of frequent conflict between him and his parents. Fortunately, his wife and children actively support him, both practically and emotionally. Despite using organic pesticides such as copper, Max still receives disparaging comments and annoyed looks from non-farming neighbors passing by for walks when he is spraying. However, his customers, who buy the products directly from the farm, know about his practices and greatly appreciate his work. Nevertheless, Max sometimes feels depressed and overwhelmed by the certification requirements and the limited time he has available. Although the cantonal farmers’ association offers assistance, he has not yet considered contacting them about his mental health. Sometimes Max thinks about giving up, feeling let down by politicians in particular. Despite his worries, he carries on every day, regularly finding joy in his work because he loves nature, his animals and having the freedom to organize his work as he sees fit. Do his colleagues feel the same way? Max occasionally overhears snippets of conversation, though he does not maintain regular contact with them. He remembers his dad talking about weekly meetings of farmers in the region, but in the age of instant messaging, there seems to be no need for regular get-togethers anymore.

**Table 1:** Science and policy recommendations through a multi-layered social resilience lens.

Multi-layered social resilience in practice		
Layer	Science recommendation	*Policy recommendation
Neighbors, Family, Friends & Colleagues	Mixed-methods approaches (qualitative and quantitative interviews) in rural areas on intervention strategies to improve social interaction and promote mutual understanding; Intervention and implementation studies to test approaches to strengthen social support; Interventions/instruments to promote farmers health resilience.	Implementation of exchange platforms between regional politicians and rural citizens (farmers, non-farmers) to promote social understanding and appreciation and the implementation of evidence (i.e., social meeting places, weekly get-togethers, etc.); (Mental) health campaigns for farmers and families.
(Urban) Society	Mixed-methods approaches (qualitative and quantitative interviews) on the factors and processes facilitating negative and positive perceptions, respectively, of agriculture, farmers and farming practices.	Educational policies and social campaigns to strengthen social appreciation of farming (i.e., promoting understanding of farming challenges in times of climate change; Understanding true costs of (organic) agricultural products; Understanding of agricultural subsidies; Understanding relevance of farmer’s protection measures).
National and Cantonal Farmer’s Association	Research on the reasons for the perceived lack of mental health support by farmers (i.e., insufficient communication, offers unsuitable for the target group); on the farmer’s associations engagement for sustainable agriculture.	Improving communication between supply and demand (i.e., mutual and transparent dialog on needs and expectations between farmers and their associations, for example on sustainable food production, Farmers who have experienced health challenges are trained as mental health multipliers to provide tailored support).
National/Cantonal Government	Policy science on the impact of climate change and sustainable farming policies on the mental health of farmers; on the impact of increased administrative burden on farmer’s motivations; Application of a holistic One-Health research approach (i.e., research on the interconnectedness between health of farmers, animals, and environment; testing interventions in improving farmers wellbeing, livestock wellbeing, and environmental wellbeing in parallel, e.g., interventions for joint vaccination of farmers and animals and pesticide-reduced farming).	Evidence-based and balanced policies toward sustainable agriculture and food production; Political and financial support for the introduction of sustainable agriculture and climate-adapted systems (i.e., livestock and plant management; implementation of One Health surveillance systems).
Overall recommendation		
Inter- and transdisciplinary science-to-policy collaboration between different study teams looking at their specific layer with the same overall goal (i.e. improved Farmers Mental Health; Sustainable Food Production – Improving multilayered social resilience). One best-practice example is the TRAPEGO project (overarching project of this article), which deals with the sustainable transformation of Swiss agriculture in order to internalize the negative external effects of pesticide use. The nature of this project necessitates an interdisciplinary and transdisciplinary approach, thereby incorporating the expertise of health, social and political scientists, agricultural scientists, environmental scientists, decision-makers, and media analysts. The objective of this collaborative effort is to examine sustainable transformation from a multitude of perspectives (i.e. health perspective).		

## Conclusion

With this perspective, drawing on non-solicited comments by Swiss farmers brought to our study, we call for research into multi-layered social resilience to address the complexity of the context that Swiss farmers operate in. Future research should examine the interaction between the various layers relevant to resilient farmers. It is imperative to comprehensively understand the multifaceted layers, their interconnection and their influence on the resilience of the agricultural community, including the identification of yet unknown layers. Addressing this can offer crucial insights for developing preventive and health-promoting interventions and targeted measures with the capacity to create pathways for a sustainable agriculture and a motivated agricultural workforce. Due to the context-specificity of the agricultural sector, this perspective focuses mainly on the Swiss context. However, the insights presented by this perspective can be of relevance for agricultural mental health research on a global scale, adjusting the different layers, enablers and risk factors to different contexts and cultures. Given the contemporary context of rapid urbanization and a concurrent decline in the agricultural workforce (21), it is imperative to prioritize the health and wellbeing of individuals responsible for global food security.

## Data Availability

The datasets presented in this article are not readily available due to the sensitive nature of the data and to ensure privacy of participants. Requests to access the datasets should be directed to nicole.probst@swisstph.ch.

## Footnotes

^1^All names were changed to protect the anonymity of the participants, citations are translated from original (German, French, Italian).

^2^Organic farming refers to the common definition of organic food production, i.e., without synthetic fertilizers or pesticides.

^3^The term ‘non-organic’ is employed in the present context as opposed to the term ‘conventional’, on the basis that the latter does not correspond to the Swiss agricultural environment, as for example the ‘proof of ecological performance’ (Ökologischer Leistungsnachweis) refers to a minimum ecological production standard.

^4^Payment system for farmers in Switzerland.

^5^Data derived from first follow-up questionnaire.
